# Tumor volume shrinkage during stereotactic body radiotherapy is related to better prognoses in patients with stage I non-small-cell lung cancer

**DOI:** 10.1093/jrr/rraa040

**Published:** 2020-07-13

**Authors:** Nam Vu, Hiroshi Onishi, Masahide Saito, Kengo Kuriyama, Takafumi Komiyama, Kan Marino, Masayuki Araya, Shinichi Aoki, Ryo Saito, Hotaka Nonaka, Satoshi Funayama, Hiroaki Watanabe, Naoki Sano

**Affiliations:** 1 Department of Radiology, University of Yamanashi, Yamanashi, Japan; 2 Department of Radiology, Hospital 175, Ho Chi Minh, Vietnam; 3 Proton Center, Aizawa Hospital, Matsumoto, Japan

**Keywords:** stereotactic body radiotherapy, lung cancer, stage I, volume change during treatment, prognosis

## Abstract

The purpose of the study was to investigate the association between tumor volume changes during stereotactic body radiation therapy (SBRT) and prognoses in stage I non-small-cell lung cancer (NSCLC). This retrospective review included stage I NSCLC patients in whom SBRT was performed at a total dose of 48.0–50.5 Gy in four or five fractions. The tumor volumes observed on computed tomography (CT) simulation and on the CT performed at the last treatment session using a CT-on-rails system were measured and compared. Then, the tumor volume changes during the SBRT period were measured and assessed for their association with prognoses (overall survival, local control, lymph node metastases and distant metastases). A total of 98 patients with a mean age of 78.6 years were enrolled in the study. The T-stage was T1a in 42%, T1b in 32% and T2a in 26% of the cases. The gross tumor volume (GTV) shrank and increased ≥10% in 23 (23.5%) and 36 (36.7%) of the cases, respectively. The 5-year local control and overall survival rates in the groups with a tumor shrinkage of ≥10% vs the group with a shrinkage of <10% were 94.7 vs 70.8% and 85.4 vs 47.6%, respectively; these differences were significant, with a *P*-value < 0.05. During a short SBRT period, the tumor shrank or enlarged in a small number of cases. A decrease of ≥10% in the GTV during SBRT was significantly related to better overall survival and local control.

## INTRODUCTION

Stereotactic body radiation therapy (SBRT) is a highly effective therapy for early-stage primary non-small-cell lung cancer (NSCLC). In this procedure, highly conformal and precise radiation doses of 48–60 Gy are delivered in three to five fractions to a lung tumor, resulting in excellent overall survival (OS) and local control rates, potentially comparable to those associated with surgery [1–6].

Initially, small tumor size changes during treatment were anticipated; however, several recent articles have shown both a decrease and an increase in inter-fractional tumor volumes [7–10]. Nevertheless, it is not known if these changes are significant and biologically meaningful. If the tumor volume changes during treatment, it may be a reflection of biological sensitivity, and therefore may be used as a predictive factor in a patient’s prognoses.

To the best of our knowledge, no studies to date have demonstrated that volumetric changes during lung SBRT are related to prognoses. Therefore, this study aimed to investigate how tumor volumes change during SBRT, and to quantify the association between tumor volume changes and prognoses, in terms of OS, local control, lymph node metastasis and distant metastasis in patients with stage I NSCLC.

## MATERIALS AND METHODS

### Patients and study design

This study was approved by the Cancer Institutional Review Board of the Office of Responsible Research Practices and written informed consent was obtained from all the enrolled patients. A retrospective study was performed on 98 consecutive stage I NSCLC patients receiving SBRT with computed tomography (CT)-on-rails imaging, between January 2006 and December 2008. A summary of the patients’ characteristics is provided in Table 1.

**Table 1:** Patient characteristics

Factors	
Number of patients	98
Age (years)	58.4–90.1 (mean 78.7)
Sex	Male, 67.3%; female, 32.7%
Performance status (PS)	PS0, 86.7%; PS1, 11.3%; PS2, 2%
Histology	Squamous cell carcinoma, 24.5%; adenocarcinoma, 50%; others, 25.5%
Stage	Stage IA, 73.5%; stage IB, 26.5%
Tumor volume (mL)	0.2–27.6 (mean 5.2)
Medical operability	Inoperable, 33.7%; operable, 66.3%

The eligibility criteria for study inclusion were: (i) histologically confirmed primary NSCLC; (ii) T1N0M0 or T2N0M0 disease according to the Union for International Cancer Control 1997 system as observed in lung CT scan images, brain magnetic resonance imaging, and bone scintigraphy or 18-fluoro-deoxyglucose positron emission tomography; (iii) greatest tumor dimension ≤5 cm; (iv) World Health Organization performance status (PS) ≤2; (v) absence of severe chronic obstructive pulmonary disease or interstitial lung disease; and (vi) absence of prior chest radiotherapy for NSCLC.

### Treatment methods

Treatments were delivered using our original unit, comprising a linear accelerator (linac) (EXL-15DP, Mitsubishi Electric, Tokyo, Japan) coupled to a CT scanner (Hi-Speed DX/I, GE Yokogawa Medical Systems, Tokyo, Japan), both of which shared a common couch [11]. The accuracy of matching between the linac isocenter and the CT image center was ≤0.5 mm. For the reproduction and maintenance of tumor position during irradiation, patients were trained in self-breath-holding at inspiration using our original breathing indicator—Abches [12]. Details on the uncertainties pertaining to the reproducibility of patient-controlled breath-hold have been previously presented [13]. Chest CT under self-breath-hold was performed for each patient and a plan was established with the help of a 3D treatment-planning computer (FOCUS, version 3.2.1, CMS, St. Louis, MO). Patients were positioned on the CT table and a skin marker for the temporary isocenter was placed using a cross-hair laser system. Clinical target volume (CTV) was equal to the gross tumor volume (GTV) delineated on CT images displayed with a window level of −300 Hounsfield units (HU) and a window width of 1700 HU. Planning target volume (PTV) was determined on CT images as the CTV plus the maximum difference of the tumor position measured on the aforementioned three repeated CT scans performed during self-breath-holding, with an additional margin of 5 mm to compensate for full internal margin including intra-session reproducibility. The first fraction of SBRT was started within 1 week after taking the CT simulation images.

In the treatment, the isocenter of the PTV was visually adjusted with CT images of 2 mm thickness taken before every radiotherapy fraction to correspond to the planned isocenter under patient self-breath-hold using the CT scanner connected to the linac. The couch was rotated 180^°^ so that the rotational center of the CT gantry corresponded to the isocenter of the linac. A more detailed account of the treatment methods has been presented previously [14, 15].

### Contouring protocol

**Fig. 1.**
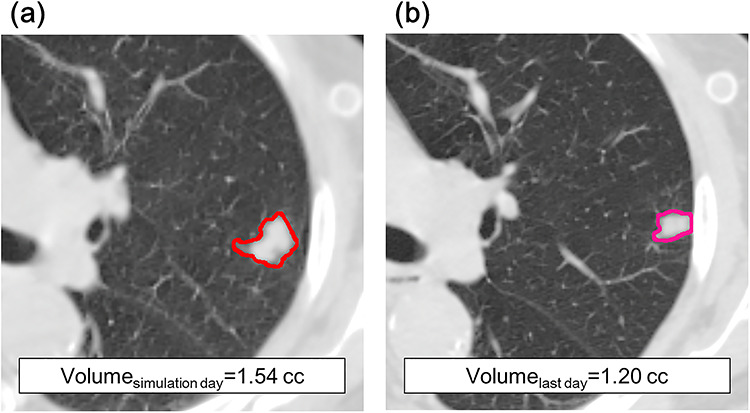
Definition of tumor volume (GTV). All CT slices are auto-contoured and verified by a physician, with an optimal threshold (−300 to 700 HU) for distinction from normal tissue. In this case, there is a small decrease in the tumor volume and diameter between the simulation day (**a**) and last treatment day (**b**).

CT images from both the simulation day and 4–5 treatment days were available for all patients. To investigate tumor volume changes, the tumors presented on the simulation day and the last treatment day were contoured. All processes were performed using MIM Maestro ver.6.6.8 (MIM Software, Inc., Cleveland, OH). The contours were semi-automatically created with the agreement of a radiation oncologist and a medical physicist. Regions out of the −300 HU to 700 HU range, such as normal lung tissue, vessels and chest walls, were automatically excluded (Fig. 1). Thereafter, GTVs were generated and calculated using this system. To distinguish ground glass attenuation from consolidation, segmentation for those cases was performed three times and the final volume was chosen by an experienced physician. For cases in which the tumor was closed or attached to the chest wall, we drew a line along the anatomical structures and repeated contouring thrice for each CT study set. The final GTV was determined from the mean value of the three contouring sets. Maximum tumor diameter was also calculated automatically by this system. Then, the tumor volume on the simulation day was used as the baseline for comparison. The same process was applied for the CT images of the last treatment day. The percentage change in GTV, on comparing the values on the simulation day and last day, was calculated using the following formula:

$$ Tumor\ volume\ change\ \left(\%\right)=\frac{Volume_{last\ day}-{Volume}_{simmulation\ day}}{Volume_{simmulation\ day}} $$

### Statistical analysis

Tumor volume changes during treatment were examined for their association with OS and local control, as well as other factors that may affect prognoses, such as age, sex, PS, tumor volume, operability, histology, tumor volume change, max standard uptake value (SUV) and the biological effective dose (BED) calculated with the linear quadratic model. OS was defined as the duration from the date of treatment to the last date of contact (death date or last follow-up date, at which point patients who were still alive were censored). Furthermore, we calculated the time to local recurrence within the same lobe. Patients who did not have recurrence or metastases were censored at the date of death or last follow-up. Patients who did not have local recurrence or distant metastases and did not die were censored at the last follow-up date.

IBM SPSS version 20 (IBM Corporation, Armonk, New York) was used for all statistical analyses. Summary statistics are provided as frequency count and percentage for categorical variables, and mean, standard deviation, median and range for continuous variables. We used a Kaplan–Meier curve to estimate the OS and time to local recurrence, and log-rank tests to evaluate the differences in time-to-event outcomes with two-sided *P*-values; *P*-values < 0.05 were considered statistically significant. In the univariate and multivariate analyses, we also calculated the hazard ratios (HRs) with their 95% confidence intervals (CIs) with a Cox proportional hazards model.

## RESULTS

### Tumor contour and tumor volume changes during SBRT

A total of 98 tumors from 98 patients were contoured. The mean tumor volume was 5.2 mL (range, 0.2–27.6 mL) on the simulation day, and 5.4 mL (range, 0.3–29.5 mL) on the last day of SBRT (day 4). The tumor volume changes are presented in Table 2. Based on the existing literature [7], a cut-off point value of 10% was used to classify the patients into two groups: Group A, tumor volume shrinkage ≥10%; Group B, tumor volume shrinkage <10% or tumor volume increased. The tumor volume changes in the two subgroups are illustrated in Fig. 1. As seen in Table 3, the BED of Group B was statistically larger than that of Group A, and there were no other significant differences between the two subgroups in terms of the baseline parameters, with a *P*-value > 0.05.

**Table 2:** Changes in tumor volume

Volume change	Criteria	Frequency	Percentage
Decrease	< −20%	0	0
	−20 to −10%	23	23.5
	−10 to 0%	24	24.5
Increase	0 to 10%	15	15.3
	10 to 20%	20	20.4
	20 to 30%	6	6.1
	>30%	10	10.2
Total	Total	98	100.0

**Table 3:** Patient characteristics by subgroup. Group A: tumor volume decrease >10%; Group B: tumor volume decrease ≤10%

Factors		Group A	Group B	*P*-value
Number of patients		23 (23.5%)	75 (76.5%)	
Age	Mean (years)	76.6	79.4	0.08
	≤75 (*n*)	6	15	
	>75 (*n*)	17	60	
Sex	Male (*n*)	14	52	0.46
	Female (*n*)	9	23	
PS	0 (*n*)	19	66	0.63
	1 (*n*)	3	8	
	2 (*n*)	1	1	
Tumor diameter, *T*	Mean (SD) (cm)	2.4 (0.95)	2.4 (0.91)	0.852
	*T* ≤ 2 cm (*n*)	9	32	
	2 cm < *T* ≤ 3 cm (*n*)	7	24	
	3 cm < *T* ≤ 7 cm (*n*)	7	19	
Tumor volume	Mean (SD) (cc)	6.2 (5.2)	4.8 (4.7)	0.22
Operability	Inoperable (*n*)	9	24	0.616
	Operable (*n*)	14	51	
Histology	Adenocarcinoma (*n*)	12	37	0.94
	SCC (*n*)	5	19	
	Others (*n*)	6	19	
SUV_max_	Mean (SD)	6.7 (6.0)	5.4 (4.7)	0.33
	≤4 (*n*)	5	24	
	>4 (*n*)	14	27	
Prescription point	Isocenter (*n*)	6	26	0.309
	ITV-D95 (*n*)	7	29	
	PTV-D95 (*n*)	10	20	
BED10 (Gy10)	Mean ± SD (Gy)	103.1 (12.5)	112.3 (13.1)	*0.003*
	≤100 (*n*)	5	1	
	>100 (*n*)	18	74	
Algorithm	Clarkson (*n*)	3	8	0.94
	Convolution (*n*)	2	6	
	Superposition (*n*)	18	61	
Duration between simulation and last SBRT fraction (day)	Median (SD)	8 (1.9)	7 (2.2)	0.59

SCC = squamous cell carcinoma; ITV = internal target volume.

### Correlation between tumor volume change and clinical outcomes

**Fig. 2.**
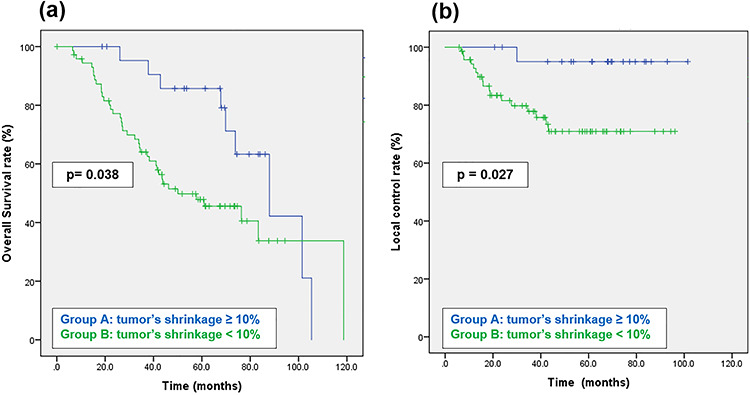
Kaplan–Meier survival curves for all patients according to tumor volume shrinkage (**a**), and Kaplan–Meier curves of local control for all patients according to tumor volume shrinkage (**b**).

The median follow-up was 74 (range, 1–119) months. The 5-year estimated outcomes were: OS, 57.9% (95% CI, 56.1–73.2%); and local control, 77.2% (95% CI, 58.6–85.2%). The 5-year local control and OS rates in the group with a tumor shrinkage >10% were significantly higher than those in the group with a tumor shrinkage <10%, at 94.7 vs 70.8% and 85.4 vs 47.6%, respectively, with a *P*-value < 0.05 across all categories (Fig. 2).

### Univariate and multivariate analyses for OS and local control

Table 4 shows the results of the statistical analysis of the prognostic factors related to OS and local control. In univariate analysis, medical operability and tumor volume change significantly (*P* < 0.05) relative to OS, whereas tumor volume, tumor volume change and BED are related to local control. However, in multivariate analysis, medical operability and tumor volume change significantly relative to OS, while only tumor volume changes are related to local control.

## DISCUSSION

The reason for the cut-off value of 10% was as follows. Based on our measurement, the percentages of patients in the three sub-groups (tumor shrinkage ≥10%, shrinkage <10% and increase in volume) are 24.5 (24 cases), 23.5 (23 cases) and 52% (51 cases), respectively. However, there were no significant statistically differences in patients’ prognoses (e.g OS, local control, lymph node metastases and distant metastases rates) between two of the sub-groups: tumor shrinkage <10% and tumor enlargement during the course of SBRT. We therefore combined patients in these both groups to form one sub-group (group B). The cut-off value of 10% was chosen based on many articles relating tumor change with SBRT treatment, and it could be simple in clinical application.

While several studies demonstrated the prognostic factors in SBRT for stage I NSCLC, Onimaru *et al*. analyzed 41 patients with stage I NSCLC treated with SBRT and reported that tumor diameter and total dose were significantly related to local control [16]. Other studies showed that tumor diameter or sex were the most significant factors affecting outcomes after stereotactic radiotherapy [17–19]. Several studies have focused on SUV in stereotactic ablative radiotherapy settings [20–23]; one of those studies—an analysis of 136 patients—found that a pretreatment SUVmax > 5.5 predicted worse recurrence and survival [23]. Another retrospective study performed by Burdick *et al*. [22] showed that pretreatment SUVmax was not predictive of regional failure, distant failure or survival.

To the best of our knowledge, this is the first study reporting that OS and local control rates differ significantly according to the tumor volume changes during SBRT. Rapid tumor volume decrease during the course of SBRT may be attributed to the tumor’s sensitivity to radiation, in particular due to tumor cell apoptosis [24]. In a study on murine tumors, adenocarcinomas tended to show higher rates of apoptosis after radiation than squamous cell carcinomas [25]. Besides, Feng *et al*. reported that a potential role of tumor-infiltrating lymphocytes (TILs) in predicting the survival of patients with completely resected stage IIIA(N2) NSCLC and the beneficial effects of TILs were more pronounced in the prediction of distant metastasis-free survival and OS in patients with squamous cell carcinoma [26]. Lymphocytes are radiosensitive and easily undergo apoptosis. Therefore, though there was no definite clinical evidence, the reason for the better prognoses of the tumor sub-group with more tumor shrinkage during SBRT might be owing to early volume loss of tumor cells or lymphocytes due to apoptosis. Further studies are mandatory to clarify this point. However, there was no statistically significant difference in OS and local control between the pathology and the tumor shrinkage in this study.

Underberg *et al*. reported that a GTV increase > 10% was observed during SBRT for NSCLC in the first 2 weeks of treatment [27] and this could be attributed by edema development due to high-dose irradiation. Tumor volume increase can adversely effect dose distribution, resulting in insufficient GTV dose coverage that might result in worse prognoses. SBRT planning should be modified according to GTV changes in such cases.

On the other hand, we also hypothesized that tumors showing a good shrinkage during SBRT could receive better dose coverage leading to better prognoses.

Our current study has some limitations. First, there might be some errors or uncertainties in the measurement of the tumor volume. The GTV obtained from semi-automatic contouring recognized only the solid parts on the CT image (expiratory phase) and did not contain spiculae and internal margins. As a result, we had to change the tumor volume manually. A 10% volume change might incidentally arise due to variations in target contouring, especially in some cases with small tumors. Another problem in the measurement was difficulty in delineating the true GTV in cases in which the tumor was located in closed proximity to other organs. The solutions to handling these cases were described in the contouring protocol section.

Second, since this is a retrospective study, there were some missing data on the cases. In particular, not all patient factors that link to OS in early-stage NSCLC have been considered and accounted for. For example, smoking history, severity of chronic obstructive pulmonary disease and the validated co-morbidity index were not included in the analysis, although those are the factors that influence OS. These points would need to be addressed in new work, including collection of patient information and data re-analysis.

Third, another notable weakness of our study is that we did not have a validation cohort of patients in order to evaluate the predictive probability of our results. Because our primary purpose was to look for the relationship between tumor change during the SBRT period, and the outcomes of patients were affected by a few different factors, our research design is not appropriate for building an optimized model for prognostic prediction.

**Table 4:** Univariate and multivariate analysis for OS and local control

Factor	Univariate analysis						Multivariate analysis					
	OS			Local control			OS			Local control		
	|HR	95% CI	*P*-value	HR	95% CI	*P*-value	HR	95% CI	*P*-value	HR	95% CI	*P*-value
Age	1.02	0.98–1.07	0.357	1.01	0.94–1.08	0.862	1.06	0.98–1.14	0.16	1.22	0.90–1.64	0.200
Sex	0.58	0.30–1.13	0.109	0.33	0.09–1.13	0.078	1.51	0.64–3.57	0.35	2.35	0.40–13.83	0.345
PS	0.98	0.48–1.99	0.959	0.35	0.05–2.36	0.183	0.67	0.18–2.57	0.56	0.13	0.00–5.23	0.281
Tumor volume	1.02	0.96–1.08	0.562	1.09	1.02–1.17	0.014	1.13	1.03–1.22	0.01	1.34	1.09–1.64	0.006
Operability	0.53	0.30–0.94	0.031	0.68	0.26–1.76	0.440	0.90	0.39–2.09	0.81	0.80	0.14–4.41	0.794
Histology	0.71	0.50–1.02	0.064	1.02	0.60–1.74	0.932	0.56	0.33–0.95	0.03	1.34	0.46–3.89	0.589
Tumor volume change	1.03	1.01–1.04	0.038	1.01	0.99–1.04	0.025	1.04	1.01–1.07	0.01	1.02	0.96–1.09	0.495
SUV_max_	0.99	0.94–1.06	0.980	1.01	0.89–1.14	0.830	0.94	0.86–1.04	0.24	0.80	0.64–1.01	0.058
BED10	4.448	0.82–24.4	0.083	32.65	1.74–61.24	0.023	0.98	0.94–1.01	0.22	0.96	0.90–1.09	0.174

Regarding the clinical benefit of examining tumor shrinkage during the SBRT period, we think this phenomenon can be used as an early predictive factor for patient response to SBRT. Though more prospective clinical studies on this finding are required, it could be suggested that the patients whose tumors do not decrease >10% or those whose tumors enlarge during the course of SBRT should be considered at higher risk of developing local recurrent or distant metastasis. As the BED of the group with the tumor shrinkage of <10% was larger than that of the other group in our results, as shown in Table 3, we cannot conceive that a higher dose of SBRT might improve the prognosis for the patients with such tumors. However, physicians should at least pay more attention to early detection or use treatment such as salvage surgery or adjuvant chemotherapy to improve survival.

Another reason for tumor volume decrease is related to cancer immunological effects. Combining SBRT and immune agents, such as checkpoint inhibitors or specific TILs against tumors, could dramatically improve the prognosis. This kind of approach is now being investigated at many centers, bringing new hope to improving the effectiveness of SBRT in the treatment of lung cancer.

## CONCLUSION

GTV shrank and increased ≥10% in 23 (23.5%) and 36 (36.7%) of cases, respectively, during SBRT. A decrease of ≥10% in GTV during SBRT is significantly related to better OS and local control.
